# Does industrial co-agglomeration promote green energy efficiency? Evidence from spatial panel data of 284 cities in China

**DOI:** 10.1007/s11356-023-31499-0

**Published:** 2023-12-29

**Authors:** Chongrong Yang, Wen Jiang

**Affiliations:** Warwick Business School, University of Warwick, CV4, 7AL, Coventry, UK

**Keywords:** Industrial co-agglomeration, Green energy efficiency, Spatial Durbin model, Super SBM model with undesirable outputs, Spatial panel data, Spatial effects

## Abstract

Industrial co-agglomeration (IC) plays a pivotal role in the development of local and adjacent green energy efficiency across 284 Chinese cities, encompassing both resource-based and non-resource-based urban centers. Based on the panel data of 284 cities in China from 2005 to 2020, this study employs spatial econometric methods to empirically assess the influence of IC and its spillover effects on green energy efficiency, employing a spatial Durbin model. Additionally, the study categorizes the 284 Chinese cities into resource-based and non-resource-based categories, utilizing spatial econometric methods to delve into the heterogeneity of their effects and spillover impacts. The key findings are as follows: (1) The average green energy efficiency across the 284 Chinese cities from 2005 to 2020 stands at 0.5834. The trend in IC indicates growth and concentration towards the central areas, increasing from 2.7396 in 2005 to 2.7658 in 2020. (2) The IC, with a coefficient of 0.0918, promotes the local green energy efficiency. (3) There are spillover effects of local IC on the green energy efficiency in adjacent areas with a coefficient of 0.2550 and an Indirect Effect of 0.4567. (4) In resource-based cities, IC positively impacts local green energy efficiency with a coefficient of 0.1056 but negatively affects green energy efficiency in adjacent areas with a coefficient of −0.1368. In non-resource-based cities, IC enhances green energy efficiency in adjacent cities with a coefficient of 0.1335. Consequently, the study offers pertinent policy recommendations aimed at improving energy efficiency in light of these findings.

## Introduction

In recent years, China’s economy has experienced rapid growth, characterized by a substantial increase in the Gross Domestic Product (GDP) value (Zhu and Lin 2022). This economic expansion, driven by the growth of heavy industries and accelerated urbanization, has resulted in a significant surge in energy demand and consumption (Jiang and Lin 2013). According to data from the China Energy Statistical Yearbook (Chinese National Bureau of Statistics 2022), China’s total energy consumption has escalated from 1469.64 million tons of standard coal (coal equivalent calculation) in 2000 to 5258.96 million tons of standard coal (coal equivalent calculation) in 2021. However, China’s economic growth has long been characterized by an extensive development model involving high input and low output, resulting in low energy utilization efficiency (Liu et al. 2017). This has led to undesirable outcomes, including environmental pollution in the form of pollutants such as wastewater and exhaust gases, which have constrained the sustainable development of China’s economy (Yang et al. 2022b). The “2022 Global Environmental Performance Index (EPI) Report” indicates that among 180 participating countries, China is ranked 160th with a score of 28.40, highlighting a significant gap in environmental performance (Environmental Performance Index 2022). To enhance green energy efficiency and promote the transformation of the industrial structure towards sustainability, the Chinese government has recently begun to emphasize the importance of industrial co-agglomeration (IC). From an international perspective, the rapid development of the regional economic integration framework represented by the Asia Pacific Economic Cooperation (APEC) provides opportunities for the Chinese government to promote sustainable urban development and gain industrial integration experience from other member countries in this region (Asia-Pacific Economic Cooperation 2017; Kakran et al. 2023). From a domestic perspective, in 2023, President Xi Jinping underscored the need for deep integration of the modern service industry with advanced manufacturing and modern agriculture, emphasizing its importance in improving the overall efficiency of the industrial system. Many cities in China, represented by Chengdu and Chongqing, have shifted their economic strategies from a primary focus on manufacturing industries to IC (Zheng and He 2022). Therefore, this paper adopts spatial econometric methods and empirically tests the efficiency of the impact of IC on green energy efficiency by constructing a spatial Durbin model (SDM). The research conclusion can provide theoretical and factual basis for the Chinese government to grasp the transformation of green industries and policy formulation.

Different from industrial agglomeration refers to the high concentration and interaction of enterprises of the same sort in a certain geographical region, as well as the continual clustering of production elements within a spatial area (Guo et al. 2023), IC is defined as the high spatial agglomeration of diverse industries in a single geographical region with upstream and downstream or horizontal links (Ellison and Glaeser Edward 1997). Previous studies have shown a spatial correlation between IC and energy efficiency. Zhang et al. (2023a) research demonstrates that manufacturing agglomeration and producer services agglomeration in the eastern and central regions of China has a significant positive impact on energy efficiency, while the western region shows no such significant positive impact. Yang et al. (2022a) research reveals a single threshold effect concerning the impact of IC on TEE. A positive effect on energy efficiency improvement only becomes evident when the degree of IC reaches a certain value. And the spatial spillover effect of IC in the eastern region is significantly higher than that in the central and western regions. Considering that the specific spatial effects generated by IC are being influenced by regional heterogeneity, and the mechanism’s influence on energy efficiency is complex, this paper chooses to employ spatial econometric instruments for further analysis.

To investigate the spatial impact of IC on energy efficiency, this study utilizes panel data encompassing 284 cities in China from 2005 to 2020, combined with the super-efficiency slack-based measure (SBM) model, entropy measurement method, and SDM to attempt to answer the following questions: (1) How can the energy efficiency of China’s 284 prefecture-level cities be reasonably measured? (2) Does IC contribute to an increase in green energy efficiency? (3) Considering the time lag, does IC exhibit geographical spillover effects and regional variation on green energy efficiency? (4) What is the impact of heterogeneity between resource-based cities (RBCs) and non-resource-based cities (NRBCs) on the spatial correlation between IC and green energy efficiency? The potential innovations and contributions of this paper are as follows: (1) This paper delves into the internal mechanism of IC’s effect on green energy efficiency using IC as the primary variable in the framework of green energy efficiency analysis. (2) By utilizing panel data from 284 Chinese cities, the SDM is utilized to scrutinize the influence of IC on green energy efficiency and its spatial spillover effect. This approach addresses the issue of spatial correlation often overlooked in conventional Ordinary Least Square (OLS) regression models, enhancing the robustness of the findings. (3) This paper investigates regional heterogeneity in the effect of IC’s effect on green energy efficiency across different regions and the heterogeneity between RBCs and NRBCs.

## Literature review

**Fig. 1:**
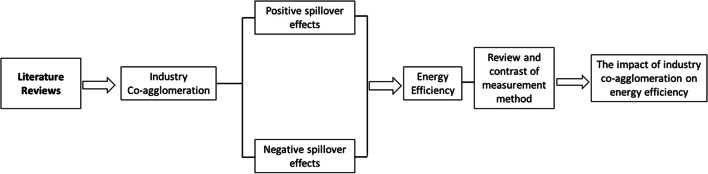
SmartArt for literature review

Industrial agglomeration is widespread worldwide. The challenges brought by the global supply chain disruption during the COVID-19 epidemic also led governments to attach importance to industry integration and agglomeration, thus promoting the industry’s resistance to external shocks (Guru et al. 2023; Wang et al. 2023). From existing literature, research on IC mainly focuses on discussing its positive and negative external impacts. Meanwhile, the pursuit of green development and the urgent need to alleviate energy poverty in developing countries have also prompted the academic community to conduct extensive research on energy efficiency field (Dagher et al. 2023). The research on energy efficiency mainly focuses on the calculation methods and influencing factors of energy efficiency. This section sorts out literature and research findings according to the roadmap design given in Fig. 1 below, in order to find existing research gaps.

### Industry co-agglomeration

Previous research on the effects of industrial agglomeration and IC on pollution (Yang et al. 2021; Zhuang et al. 2021), labor productivity (Ushifusa and Tomohara 2013; Yang 2016), innovation ability (Guo et al. 2023; Yang et al. 2022b), and green economic performance (Xu and Yang 2019; Yuan et al. 2020) have been conducted. However, there is no consensus on the impact of industrial agglomeration and IC on the local economy and ecological environment. On the one hand, industrial agglomeration and IC can improve production efficiency, promote green innovation, and alleviate environmental pollution through technology and knowledge sharing effects, economies of scale, and competition encouragement mechanisms (Morrison Paul and Siegel 1999; Xie and Li 2021). A study on the synergistic agglomeration of production services and manufacturing industries shows that market-driven IC can not only greatly improve local ecological environment pollution control but also significantly promote ecological environment pollution control in the surrounding region due to the spatial spillover effect (Yang et al. 2021). On the other hand, industrial agglomeration caused by government intervention may lead to a “free rider” effect, which refers to the various sharing effects of industrial agglomeration that deepen the inertia and dependence of some enterprises and affect the innovation ability and productivity of the companies (Cheng 2016; Han et al. 2018). Meanwhile, the overcrowding effect caused by excessive industrial agglomeration may also lead to imbalanced resource allocation and uneconomic scale (Brülhart and Mathys 2008; Chen et al. 2018; Fontagné and Santoni 2019).

### Energy efficiency

Energy efficiency measurements can be categorized as either single factor or total factor energy efficiency (TEE). The single energy efficiency measurement, which is used to measure the proportional relationship between the energy input and the effective output, may exaggerate the effect of energy inputs on economic output and cannot accurately reflect energy efficiency (Bu et al. 2022). As a result, TEE, which takes into account the effects of mutual substitution between various production factors and structural changes in the production process on energy efficiency (Liu et al. 2020), was considered that the impact of the interaction between various input factors on energy efficiency had been fully reflected. Thus, TEE was adopted as the indicator to reflect green energy efficiency in this paper.

The existing measurement methods for energy efficiency mainly include the stochastic frontier analysis (SFA) method and the data development analysis (DEA) method. The SFA method needs to be given different production functions and assumptions when measuring energy efficiency, and the deviation of the function form will affect the measurement effect (Wu et al. 2014). In contrast, as a non-parametric method, DEA can handle multiple inputs and outputs simultaneously. There is no need to estimate the cost and production function and the expected output and unexpected output can be distinguished (Mardani et al. 2017). Given the advantages of the DEA model, a large number of scholars have chosen the DEA method to estimate energy efficiency (Jebali et al. 2017; Wang et al. 2012; Zhang and Chen 2022).

Numerous academics have studied the variables that affect energy efficiency; these studies include environmental regulation (Pan et al. 2019), energy endowment (Wang et al. 2022b), industrial structure (Yu 2020), technological advancement (Li and Lin 2018), and others. The research on the impact of IC on energy efficiency is still in its initial stage. The impact mechanism of IC on energy efficiency is mainly summarized from empirical researches on the impact of industrial agglomeration on energy efficiency. There are mainly three aspects. The first view speculates that IC is beneficial for energy efficiency because it helps form the scale economy effect and the resource sharing effect, which reduce the production cost and unit energy consumption (Yang et al. 2021). The second view speculates that IC accompanied by low coupling coordination is likely to result in a squeeze on the industrial chain, violent competition, and inefficient equilibrium, which may block energy efficiency (Zhu et al. 2022). The third viewpoint holds that there is a non-linear relationship between industrial agglomeration and energy efficiency. Some factors brought about by IC, such as complementary effects and technique effects, may bring positive impacts, while scale effects and crowding out effects may bring negative impacts on energy efficiency (Li et al. 2019; Wu et al. 2021).

The existing research provided a valuable theoretical foundation for this study, but it is not without its limitations, which this research aims to address: (1) Previous research has primarily focused on analyzing the external effects of single industrial agglomerations, while there has been relatively less discussion regarding the spatial effects of IC. (2) There are discrepancies in the methods used for efficiency calculation. Many energy efficiency models, such as the SFA model and non-parametric DEA accounting method, tend to overlook environmental factors such as resource input and pollutant emissions. (3) The examination of energy efficiency from the perspective of IC is still in its early stages. Few prior studies in China have employed spatial econometric methods to explore the spatial spillover effects of IC on energy efficiency. Additionally, these studies have often relied on provincial panel data, failing to account for variations among different cities within the province. To overcome these limitations, this paper employs the panel data of 284 cities in China from 2005 to 2020 and uses the super-efficiency SBM model with undesirable outputs to calculate the TEE of all cities in China. Building on this foundation, this paper employs the SDM to empirically discuss the impact of IC between the manufacturing and service industries on TEE and its spatial spillover effect.

## Method and data

### Model construction

#### Setting spatial weight matrix (*W*)

In spatial econometrics, the spatial weight matrix is crucial for the spatial econometric model. There are three types of the spatial weight matrix, which are the economic weight matrix (*W*_1_), the 0–1 weight matrix (*W*_2_), and the geographic distance weight matrix (*W*_3_), used in this study.

1 $$W_1=\left\{\;\begin{array}{c}\frac1{\left|\overline{GDP_i}-\overline{{GDP}_j}\right|}\;i\neq j\\0\;i=j\end{array}\right.\;\text{where},\text{i}=1\dots\text{n};\;\text{j}=1\dots\text{n};\;\text{n}=284$$

The Formula (1) is the expression of the economic weight matrix. The $\overline{GDP_i}$ and $\overline{GDP_j}$ are the average GDP in cities *i* and *j*, respectively, and *n* means the number of cities.

2 $${W}_2=\left\{\begin{array}{c}1\ if\ i\ and\ j\ and\ adjacent\ \\ {}0\ if\ i\ and\ j\ are\ nonadjacent\end{array}\right.$$

The Formula (2) is the expression of the 0–1 spatial weight matrix, which is used to represent the relationship of adjacency between cities. If two cities’ administrative boundaries are not adjacent, the value is 0; if not, it is 1.

3 $$W_3=\left\{\begin{array}{c}\frac1{d_{ij}^2}\;i\neq j\;\\0\;i=j\;\end{array}\right.\;\mathrm{where},\;\text{i}=1\dots\text{n};\;\text{j}=1\dots\text{n};\;\text{n}=284$$

The Formula (3) is the expression of the geographic distance weight matrix. The *d*_*ij*_ is the distance between cities *i* and *j*.

#### Test of spatial correlation

The existence of spatial correlation is the prerequires for conducting the spatial econometric model. Moran Index is the method that is usually used in the test of spatial correlation to verify its existence (Anselin 1995). The Global Moran Index and Local Moran Index are included in the Moran Index.

4 $${Global\ Moran}^{\prime }s\ {I}_{it}=\frac{\sum_{i=1}^n\sum_{j=1}^n{W}_{ij}\left({x}_i-\overline{x}\right)\left({x}_j-\overline{x}\right)}{S^2\sum_{i=1}^n\sum_{j=1}^n{W}_{ij}}$$

The spatial correlation of variables in different areas is determined by Global Moran Index, and the above Formula (4) shows the calculation formula for it, where *W*_*ij*_ is the spatial weight matrix and *S*^2^ is the sample variance. The range of value of Moran’s I is from −1 to 1. If the value is larger than 0, there is a positive spatial correlation. If the value is below 0, it is a negative spatial correlation. However, if the value tends to 0, it means there is no correlation in space.

5 $${Local\ Moran}^{\prime }s\ {I}_{it}=\frac{\left({x}_i-\overline{x}\right)\sum_{j=1}^n{W}_{ij}\left({x}_j-\overline{x}\right)}{\frac{\sum_{i=1}^n{\left({x}_i-\overline{x}\right)}^2}{n}}$$

The spatial agglomeration near a region is examined by Local Moran Index, and the expression of it is Formula (5). The Local Moran Index is usually illustrated by the Moran Scatter Diagram. There are four quadrants in the diagram to show four spatial correlation relationships between the sample area and surrounding areas. The first quadrant and third quadrant show the “high-high” (HH) and “low-low”(LL) relationships, which means that there is a high (low) value in a region, the areas near this region also contain a high (low) value to show the positive spatial correlation relationship between them. The second and fourth quadrant indicates the “high-low” (HL) and “low-high” (LH) means the negative spatial correlation relationship that a region is high (low) value, but the areas near that region is low (high) value.

#### Spatial econometric model established

According to Du et al. (2022) and Yu (2021), we need to construct the benchmark panel model that examines the impact of IC on TEE first. The information is shown in the following Formula (6):

6 $${TEE}_{it}=\alpha +\beta {IC}_{it}+\gamma {X}_{it}+{\mu}_i+{\delta}_t+{\epsilon}_{it}$$

*TEE*_*it*_ is the explained variable in Formula (6), representing the TEE of the city *i* in year *t*. *IC*_*it*_ is the core explanatory variable, signifying the IC of the city *i* in year *t*. *X* is a set of control variables. *μ*_*i*_ represents the fixed effect, *δ*_*t*_ stands for the time effect, and *ϵ*_*it*_ represents the random disturbance term. The regression coefficients for the intercept term, the core explanatory variable, and the control variable are denoted by *α*, *β*, and *γ*, respectively.

The benchmark panel model does not take the spatial correlation of variables into account. Considering TEE has a spillover effect geologically by nature and the spatial econometric model can avoid ignoring the spatial correlation between cities, this paper undertakes an analysis by developing a spatial econometric model (Anselin 2010). Spatial econometric models mainly include the following three types: Spatial Lag Model (SAR), Spatial Error Model (SEM), and SDM (Anselin and Getis 1992; Elhorst 2014), which are shown in the following Formula (7), Formula (8), and Formula (9) separately.

7 $${TEE}_{it}=\rho \sum {W}_{ij}\times {TEE}_{it}+\beta {IC}_{it}+\gamma {X}_{it}+{\epsilon}_{it}$$

8 $${TEE}_{it}=\beta {IC}_{it}+\gamma {X}_{it}+{\epsilon}_{it},{\epsilon}_{it}=\delta \sum {W}_{ij}\times {\epsilon}_{it}+{\mu}_{it}$$

9 $${TEE}_{it}=\rho \sum {W}_{ij}\times {TEE}_{it}+\beta {IC}_{it}+\gamma {X}_{it}+\eta \sum {W}_{ij}\times {IC}_{it}+\theta \sum {W}_{ij}{\epsilon}_{it}\times {X}_{it}+{\epsilon}_{it}$$

where *ρ* represents the spatial regression coefficient of TEE; *δ* represents the spatial error coefficient, *η* is the spatial regression coefficient of IC, *θ* represents the spatial regression coefficient of the control variable. *W* stands for spatial weight matrix. Formula (7) is the SAR model, which includes only the spatial lag term of the TEE. This indicates that the SAR model only considers the spatial spillover effects of the explained variables and does not consider the spatial spillover effects of the core explanatory variables and other variables. Formula (8) is the SEM model, reflecting the spatial spillover effect of random disturbance terms. Compared to the SAR and SEM models, the SDM represented by Formula (9) contains the spatial regression coefficients of both the TEE and the IC, demonstrating that the SDM model can incorporate both the explained and explanatory variables into the spatial correlation analysis. Therefore, the spatial spillover effect of IC on TEE is analyzed using the SDM model in this paper.

### Variables

#### Explained variable: total factor energy efficiency

The super-efficiency SBM model with undesirable outputs will be used in this paper to estimate the TEE of 284 Chinese cities between 2005 and 2020. Compared to other DEA models, the super-efficiency SBM model with undesirable outputs can address the issue that the traditional radial DEA model does not include slack variables in efficiency measurement and account for the impact of undesirable outputs on energy efficiency by adding unexpected outputs to the output (Tone 2002, 2004). Its basic principle is as follows.

Assume there are *n* decision-making units (DMU) and that each DMU contains three sets of factors: input set *m*, desirable output factor set *S*_1_, and undesirable output factor set *S*_2_. Then, the input, desired output, and undesirable output vector sets can be written as $X=\left[{x}_1,{x}_2\cdots, {x}_n\right]\in {\mathbb{R}}_{+}^{m\times n}$, ${Y}^g=\left[{y}_1^g,{y}_2^g,\cdots, {y}_n^g\right]\in {\mathbb{R}}_{+}^{s_1\times n}$, and ${Y}^b=\left[{y}_1^b,{y}_2^b,\cdots, {y}_n^b\right]\in {\mathbb{R}}_{+}^{s_2\times n}$, respectively. Based on this, the production possibility set (PPS) can be expressed as follows: *PPS* = {(*x*, *y*^*g*^, *y*^*b*^)|*x* ≥ *Xλ*, *y*^*g*^ ≤ *Y*^*g*^, *y*^*b*^ ≥ *Y*^*b*^, *λ* ≥ 0}, in which *λ* is the non-negative intensity vector. According to Tone (2004), Formula (10) demonstrates the algorithm for super-efficiency SBM with undesirable outputs.

10 $${\displaystyle \begin{array}{cc}& \min \rho =\frac{1+\frac{1}{m}\sum_{i=1}^m\frac{s_i^{-}}{x_{ik}}}{1-\frac{1}{s_1+{s}_2}\left(\sum_{r=1}^{s_1}\frac{s_r^g}{y_{rk}^g}+\sum_{t=1}^{s_2}\frac{s_t^b}{y_{tk}^b}\right)}\\ {}\textrm{s}.\textrm{t}.& \sum_{j=1,j\ne k}^n{x}_{ij}{\lambda}_j-{s}_i^{-}\le {x}_{ik}\\ {}& \sum_{j=1,j\ne k}^n{y}_{rk}{\lambda}_j+{s}_r^g\ge {y}_{rk}^g\\ {}& \sum_{j=1,j\ne k}^n{y}_{tj}{\lambda}_j-{s}_t^b\le {y}_{tk}^b\\ {}& \sum_{j=1,j\ne k}^n{\lambda}_j=1\\ {}& \lambda \ge 0,{s}_i^{\textrm{g}}\ge 0,{s}_r^b\ge 0,{s}^{-}\ge 0\\ {}& i=1,2,\cdots m;r=1,2,\cdots {s}_1;t=1,2,\cdots {s}_2;j=1,2,\cdots n\left(j\ne k\right)\end{array}}$$

In Formula (10), the slack variables of inputs, desirable outputs, and undesirable outputs are denoted by ${s}_i^{-}$, ${s}_r^g$, and ${s}_r^b$, respectively; *λ*_*j*_ is the weight vector; *ρ* is the efficiency value of DMU. When *ρ*≥ 1, DMU is efficient. If *ρ* < 1, the DMU is inefficient, and the input-output mechanism requires improvement.

According to Du et al. (2022) and Guo et al. (2018), we have constructed an input-output indicators system and measured the TEE in 284 cities using the super-efficiency SBM model with undesirable output. The contents of the indicators are detailed in Table 1.

**Table 1:** Input-output indicators for total factor energy efficiency

Indicator type	Indicator name	Indicator description/unit
Input	Labor	Number of employees at the end of the year (10 thousand people)
	Capital	Fixed assets of the whole society (10 thousand yuan)
	Energy	Total urban electricity consumption (100 GW)
Expected output	GDP	Gross domestic products of the cities (100 million yuan)
Unexpected outputs	Waste water	The volume of industrial waste water discharged (10,000 tons)
	Waste gas	The volume of industrial sulfur dioxide emission (10,000 tons)
	Smoke (powder) dust	Industrial smoke (powder) Dust emission (10,000 tons)

The input indicators include labor input, capital input, and energy input: (1) labor input: The optimal input of the labor force should consider both labor force quality and labor time. Due to the dearth of pertinent statistical data, this article evaluates labor input based on the number of employees in each city at the end of the year. (2) Capital input: Due to the lack of official capital stock survey data, this paper employs the perpetual inventory method to measure the total social fixed assets investment in each city. The specific calculation equation is *K*_*i*, *t*_ = (1 − *δ*)*K*_*i*, *t* − 1_ + *I*_*i*, *t*_ where *K*_*i*, *t*_ is the capital stock of period at city i, *I*_*i*, *t*_ is the fixed assets investment of period *t* at city *i*, and *δ* is the depreciation rate. The depreciation rate of fixed assets is set at 10.96% to eliminate the impact of price fluctuation factors (Yu and Shen 2020). (3) Energy input: Considering that fossil fuels are still the primary source of energy for power generation in China (Tang et al. 2018), the total electricity consumption can be reflective of energy consumption to a certain extent. Therefore, the total electricity consumption of each city is used as the energy input index in the paper.

The outputs can be divided into the expected outputs and the undesirable outputs: (1) desirable outputs: the GDP of each city is used as an indicator of expected output. (2) With the coexistence of industrialization and urbanization in China, ecological factors are increasingly becoming a limiting factor for improving TEE. This paper takes the industrial waste water discharged, industrial sulfur dioxide emission, and industrial smoke (powder) dust emission as unexpected outputs of TEE.

#### Explanatory variable: industry co-agglomeration

The core explanatory variable of this paper is the IC between the manufacturing and service industries. Following the study of Li et al. (2019) and Peng et al. (2022), the IC exponent refers to the measurement method and is constructed as follows:

11 $$LQ{M}_{int}=\frac{\frac{E_{nit}}{E_{nt}}}{\frac{E_{it}}{E_t}}$$

12 $$LQ{S}_{ipt}=\frac{\frac{E_{pit}}{E_{pt}}}{\frac{E_{it}}{E_t}}$$

13 $${LQA}_{it}=\left(1-\frac{\left|{LQM}_{int}-{LQS}_{ipt}\right|}{LQM_{int}+{LQS}_{ipt}}\right)+\left|{LQM}_{int}+{LQS}_{ipt}\right|$$

In Formula (11), Formula (12), and Formula (13), *LQM*_*int*_ represents the location entropy exponent of the manufacturing industry in the city *i* during period *t*; *LQS*_*ipt*_ represents the location entropy exponent of the service industry in the city *i* during period *t*. *E*_*nit*_ and *E*_*nt*_ represent the number of employees in the manufacturing industry in the city *i* and the total number of employees in the manufacturing industry in the country, respectively; *E*_*pit*_ and *E*_*pt*_ represent the number of employees in the service industry in the city *i* and the total number of employees in the service industry in the country, respectively. *E*_*it*_ and *E*_*t*_ represent the total number of employed people in the city *i* and the total number of employed people in the country separately. *LQA*_*it*_ is the co-agglomeration exponent of the manufacturing industry and service industry in the city *i* during period *t*. The greater the value of *LQA*_*it*_, the greater the co-agglomeration of the two industries in this city, and vice versa.

#### Control variable

**Fig. 2:**
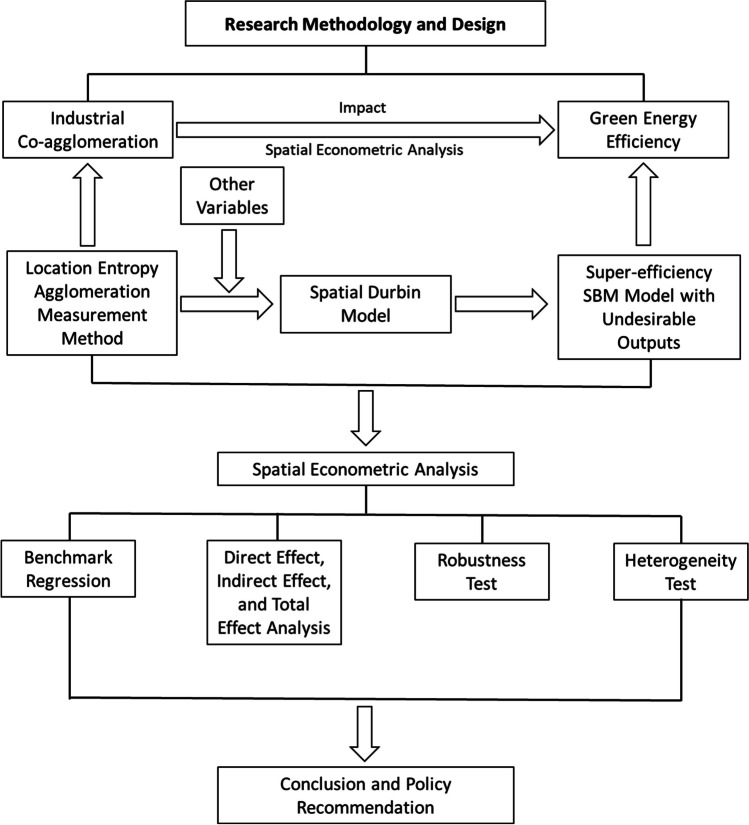
Research roadmap

In addition to IC, many factors can affect the TEE. The following control variables are added in the regression process: (1) level of economic development (ED): This paper uses the per capita GDP of each city to measure the level of economic development. (2) Level of financial development (FD): This paper uses the ratio of deposits and loans of financial Institutions at year-end to the GDP as a measure of the level of financial development. (3) Level of opening up (*F*): This paper employs the ratio of foreign direct investment to GDP as a measure of the level of opening up. (4) Fiscal expenditure (FE): This paper uses the ratio of local general public budgeting expenditure to the GDP as a measure of fiscal expenditure. (5) Level of human capital (HC): This paper employs the logarithm of the ratio of registered students in high education institutions to the population as a measure of the level of human capital per capita. (6) Level of transport infrastructure (TI): This paper uses the ratio of urban road mileage to population as a measure of the level of transport infrastructure. To summarize, the research roadmap reflecting the research methodology and design of this paper is shown in the following figure (Fig. 2). The conceptual framework and evaluation of key indicators in this article lay the foundation for understanding the differences in TEE and IC levels at the urban level in China, as well as the spatial effects of IC levels on energy efficiency. And provide a reference for formulating relevant industrial integration policies and urban differential development policies, thereby promoting energy efficiency improvement, industrial structure transformation, and sustainable development.

### Study area, data source, and statistical description of samples

**Fig. 3:**
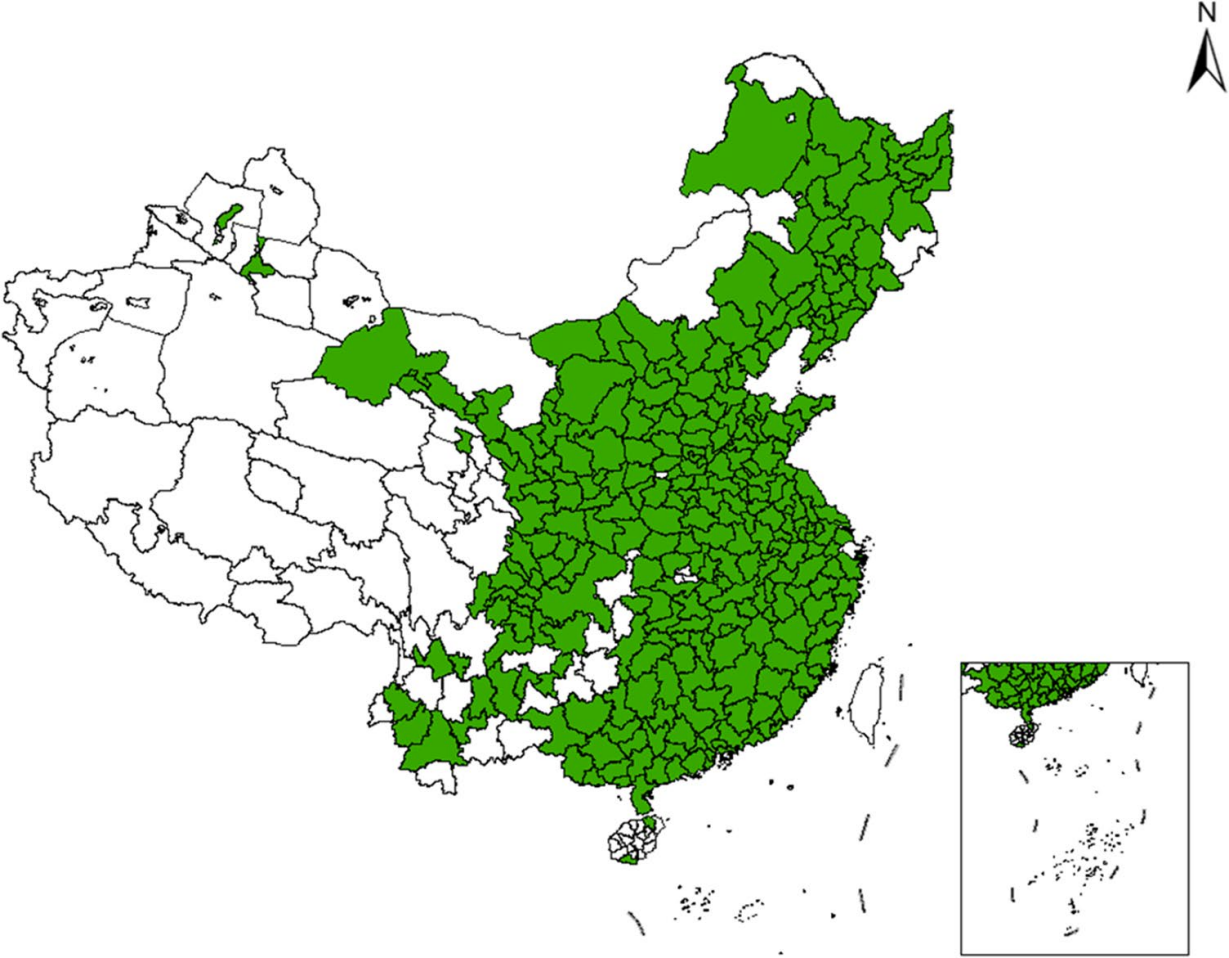
Study area

As research samples, 284 prefecture-level cities were selected from 333 prefecture-level administrative regions in China. As shown in Fig. 3, these 284 cities include the capital cities of China’s 31 provincial administrative regions (excluding Hong Kong, Macao, and Taiwan), as well as tourist cities, industrial cities, etc., which are widely distributed across China’s eastern, central, and western regions.

The original data of all variables are from the official data published in the 2005–2020 China Urban Statistical Yearbook and the statistical yearbooks of relevant cities and provinces. This paper uses the linear interpolation method to supplement a small number of missing values in the sample data. The statistical description of the study samples is shown in Table 2 below.

**Table 2:** Statistical description of the sample

Variable	Obs	Mean	Std. dev.	Min	Max
Total factor energy efficiency	4544	0.583	0.522	0.151	11.183
Industry co-agglomeration	4544	2.751	0.234	1.246	3.118
Level of economic development	4544	10.398	0.773	4.595	15.675
Level of financial development	4544	0.902	0.542	0.075	5.305
Level of opening up	4544	0.018	0.020	0	0.212
Fiscal expenditure	4544	1.796	1.003	0.426	10.268
Level of human capital	4544	10.377	1.428	3.829	13.922
Level of transport infrastructure	4544	3.161	1.901	−0.34	19.968

## Results

### Results of total factor energy efficiency and industrial co-agglomeration

#### Results of total factor energy efficiency

**Fig. 4:**
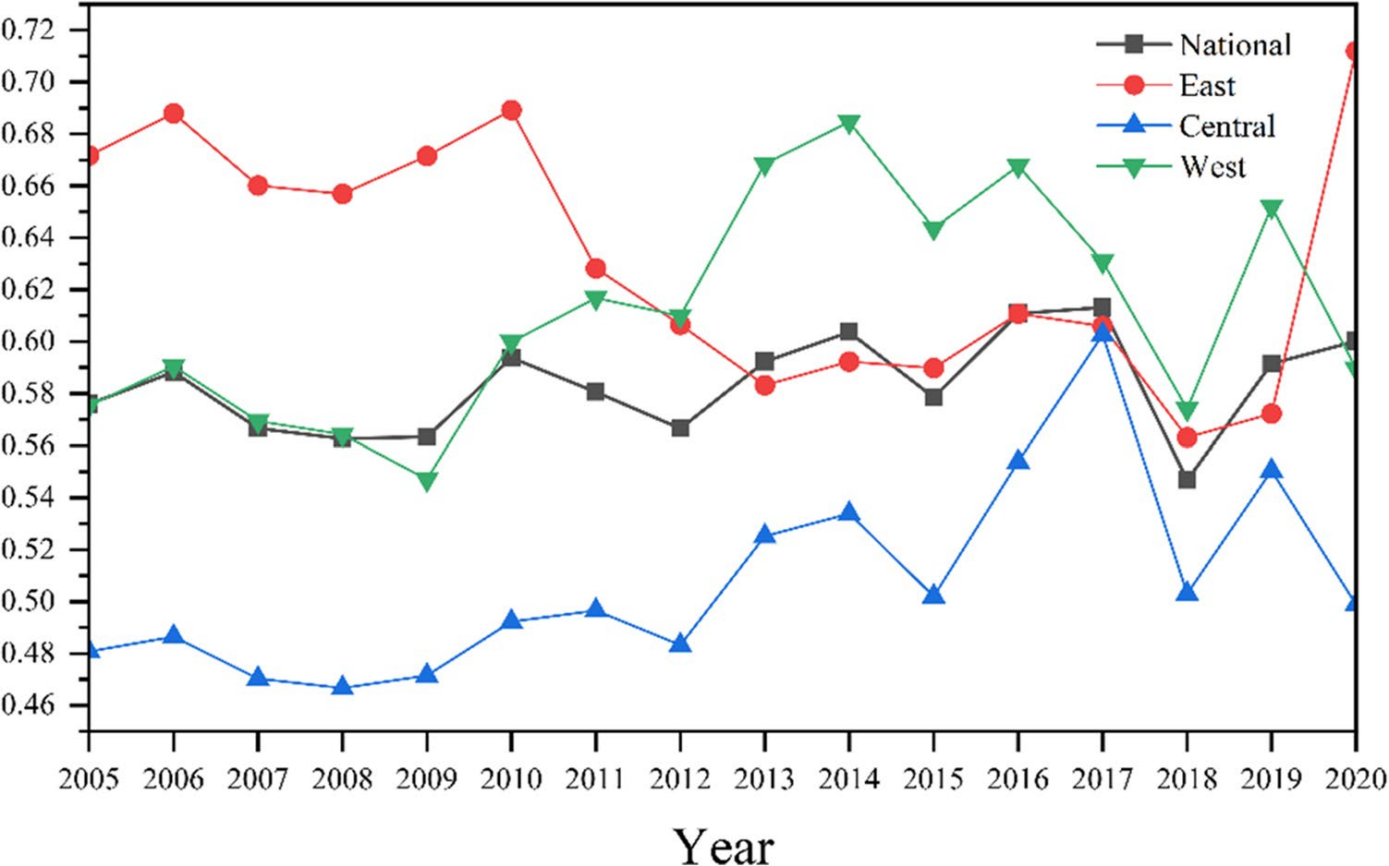
Temporal and regional characteristics of TEE

The TEE for 284 cities in China from 2005 to 2020 is calculated based on the super-efficiency SBM as well as the undesirable outputs model. Figure 4 shows TEE’s temporal and regional characteristics from 2005 to 2020. The average value of the national TEE from 2005 to 2020 is 0.5834, which is less than 1, and this means that the TEE in China is relatively low and can be improved further.

From the view of temporal characteristics, the overall trend of National TEE in 284 cities in China from 2005 to 2020 is fluctuating, which is “falling first and then rising.” The mean of TEE increased from 0.5760 to 0.6001 over the period spanning 2005 to 2020. This means the overall energy efficiency is improving continuously, and the emphasis on energy efficiency is increasing.

From the view of regional characteristics, the 284 cities in China are separated into East, Central, and West to discuss the further regional differences. The mean of TEE in East, Central, and West from 2005 to 2020 are 0.6313, 0.5074, and 0.6115, respectively. The average TEE in the West is close to the National average TEE, and that in the East and West performs better than the National average, but that in the Central is not. Moreover, the Central TEE performs poorer than the National TEE in most years. Thus, the regional characteristics of TEE are that TEE in East > that in West > that in Central.

#### Results of industry co-agglomeration

**Fig. 5:**
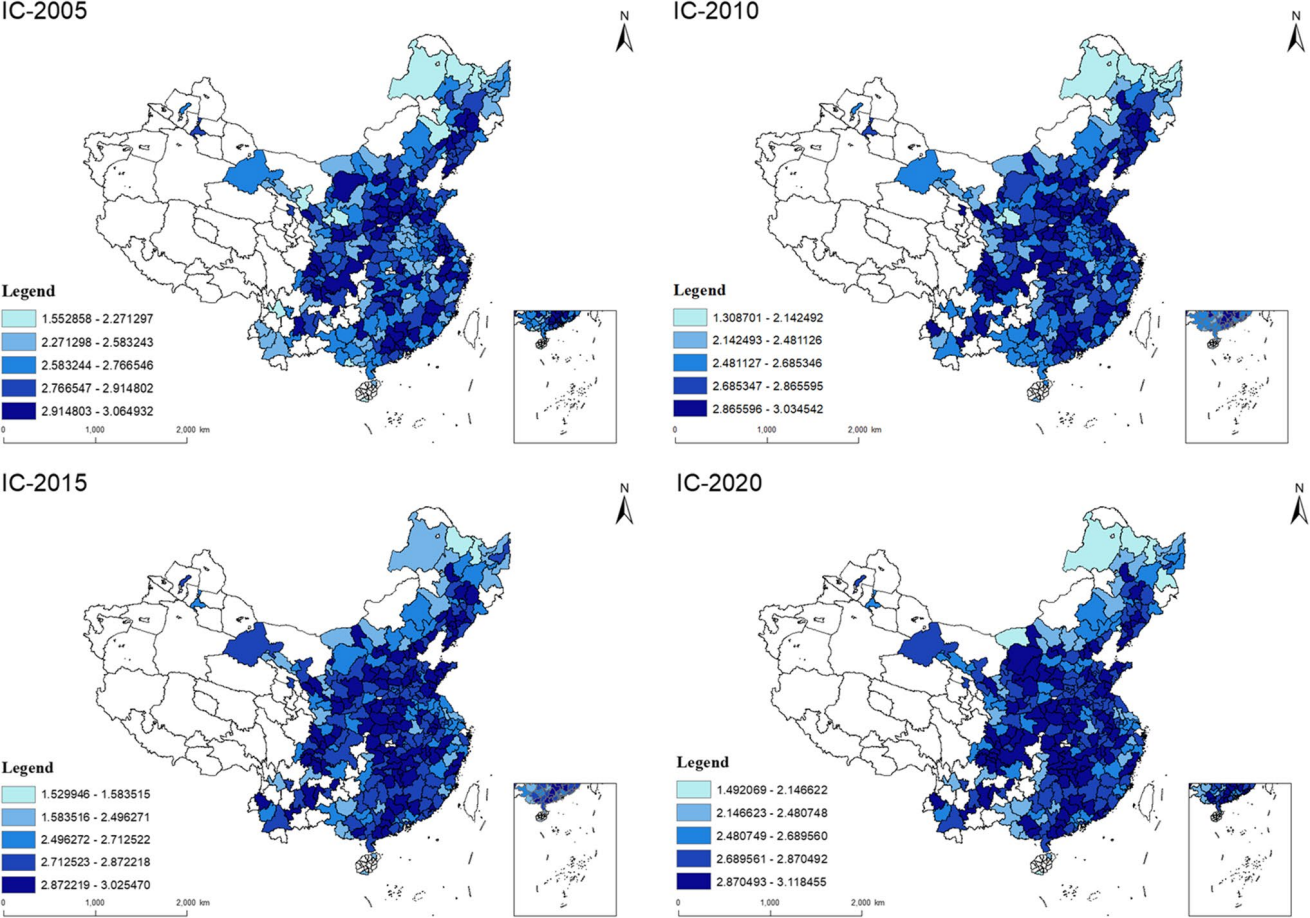
Spatial characteristics of IC

The calculation results of 284 Chinese cities’ IC from 2005 to 2020 are shown in Fig. 5. In 2005, the coastal cities in the eastern part of China had a higher IC than the cities in the western part, and central cities had lower IC than the western cities. However, in 2020, most cities have higher IC than the eastern and western cities, although the IC in the East and West are also strengthened, which also means most Chinese cities in 2020 have higher IC than in 2005. Therefore, there is a clear trend from 2005 to 2020 that the IC in China is growing and concentrating on the central part.

### Results of the spatial correlation test

The results of the Global Moran Index for TEE and IC are shown in Table 3. The most Global Moran Index from 2005 to 2020 are positive and significant at the levels of significance 1% and 5%. Although the result for TEE in 2020 is not significant at a significance level of 10%, it is acceptable for overall test results relatively (Zhao et al. 2021). Thus, there is a positive spatial correlation between TEE and IC.

**Table 3:** Results of Global Moran’s I

Years	TEE		IC	
	Global Moran’s I	*z*-value	Global Moran’s I	*z*-value
2005	0.007***	2.572	0.046***	9.71
2006	0.007***	3.667	0.055***	11.425
2007	0.006***	3.334	0.061***	12.607
2008	0.005***	2.448	0.058***	12.1
2009	0.006***	2.62	0.053***	11.219
2010	0.004**	2.227	0.057***	11.927
2011	0.006***	2.571	0.045***	9.538
2012	0.013***	3.81	0.043***	9.178
2013	0.01***	3.068	0.034***	7.34
2014	0.02***	5.436	0.04***	8.515
2015	0.016***	4.613	0.041***	8.826
2016	0.017***	4.652	0.038***	8.054
2017	0.01***	2.633	0.053***	11.074
2018	0.021***	5.037	0.086***	17.582
2019	0.02***	4.624	0.081***	16.601
2020	−0.001	0.461	0.051***	10.681

*, **, and *** represent the significance at 10%, 5%, and 1%, respectively

**Fig. 6:**
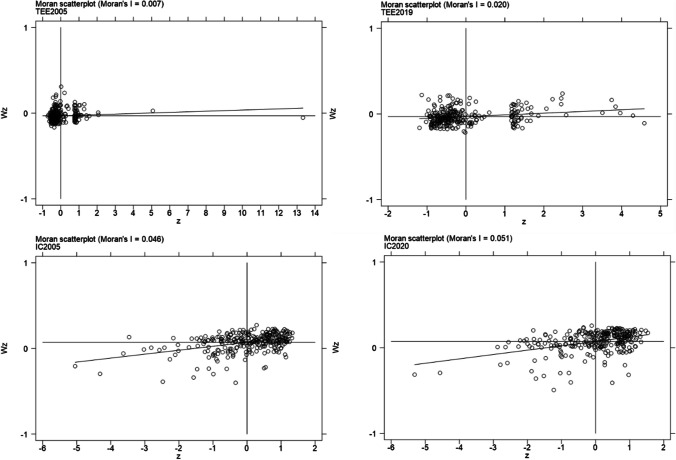
Local Moran Scatter Diagrams of TEE and IC

According to the Moran Scatter Diagram and results of the Moran Index in Table 3 and Fig. 6, the spatial distribution for TEE and IC is not randomly distributed. On the Moran Scatter Diagram, most cities are located on the first quadrant and third quadrant with the characteristics of “high-high” and “low-low,” which demonstrated the positive spatial correlation in TEE and IC. Thus, the following analysis conducted by the spatial econometric model is reasonable.

### Results of benchmark regression

It is essential to utilize LR, Wald, and Hausman test to find a reasonable model to conduct further analysis (LeSage and Pace 2010; Wang et al. 2022a). Table 4 shows the results of LR, Wald, and Hausman tests under Spatial Matrix W3. The SDM model cannot convert to SAR or SEM model because the *p*-values of LR and Wald tests are significant at the significance level of 1%. Hence, the SDM model is more reasonable to use in further analysis. Moreover, the result of the Hausman test is also significant at the level of 1%, so it is more suitable to use a fixed effect model rather than a random model in further analysis.

**Table 4:** The results of LR, Wald, and Hausman tests

LR SAR	LR SEM	Wald SAR	Wald SEM	Hausman test
29.56	32.73	29.65	31.46	34.24
(0.0001)	(0.0000)	(0.0001)	(0.0001)	(0.0000)

*p*-value in the parentheses

Table 5 shows the estimated results of benchmark regression. In Table 5, Columns (1) and (2) are the regression result based on the OLS model with fixed effect and random effect, respectively. As mentioned before, from the result of the Hausman test, the regression result in Column (1) is superior rather than Column (2). The coefficient of IC is 0.0872, and it is positive and significant at a significance level of 5%. This means IC has a significant positive effect on TEE, which an increase of 1% for the IC causing a 0.0872% increase in the TEE. For the variables, the coefficient of FD is negative and significant at the level of 1% to indicate that the development FD is not beneficial for the improvement of TEE. The coefficients of *F* with significance at a level of 10%, FE and TI are positive, as well as that of FE and TI are significant at the level of 1%. The results represent that the increase in the *F*, FE, and TI cause positive impacts on TEE. The coefficients of ED and HC are not positive and negative, respectively, and both are not significant.

**Table 5:** The results of benchmark regression

TEE	(1)	(2)	(3)	(4)	(5)
	OLS-FE	OLS-RE	SAR	SEM	SDM
			W3	W3	W3
IC	0.0872**	0.0087	0.0893**	0.0895**	0.0918**
	(1.972)	(0.208)	(2.089)	(2.087)	(2.145)
ED	0.0024	0.0096	0.0080	0.0033	0.0239
	(0.183)	(0.792)	(0.622)	(0.265)	(0.973)
FD	−0.0879***	−0.0670***	−0.0870***	−0.0834***	−0.1270***
	(−3.990)	(−3.257)	(−4.082)	(−3.818)	(−4.871)
*F*	0.7690*	1.0995***	0.7304*	0.7638**	0.4606
	(1.934)	(2.869)	(1.897)	(1.98)	(1.14)
FE	0.0360***	0.0411***	0.0355***	0.0348***	0.0332**
	(2.896)	(3.665)	(2.954)	(2.867)	(2.557)
HC	−0.0035	−0.0085	−0.0039	−0.0047	0.0037
	(−0.219)	(−0.695)	(−0.254)	(−0.307)	(0.238)
TI	0.0244***	0.0158**	0.0236***	0.0234***	0.0229***
	(3.435)	(2.467)	(3.433)	(3.368)	(3.186)
*W* × IC					0.7298**
					(2.030)
*W* × ED					0.1121*
					(1.690)
*W* × FD					0.2532***
					(2.944)
*W* × *F*					−7.6657
					(-1.62)
*W* × FE					−0.3113***
					(−2.794)
*W* × HC					0.0145
					(0.078)
*W* × TI					−0.0197
					(−0.735)
Constant	0.2791	0.4656***			
	(1.551)	(3.012)			
*ρ* or *λ*			−0.3104*	−0.1139	−0.4588**
			(−1.878)	(−0.703)	(−2.237)
Observations	4544	4544	4544	4544	4544
Fixed effect	Yes	Yes	Yes	Yes	Yes
Time effect	Yes	Yes	Yes	Yes	Yes
Log-likelihood			−981.8520	−983.4365	−967.0734

*, **, and *** represent the significance at 10%, 5%, and 1%, respectively; *z*-value in the parentheses

Columns (3) to (5) in Table 5 are the regression result of SAR, SEM, and SDM models, respectively. The assumption of the spatial econometric model is that there are spatial correlations among different areas, so the assumption of OLS cannot be applied to this case. Thus, the Maximum Likelihood Estimation method is more reasonable to be utilized in the spatial econometric model.

From Columns (3) to (5), the results of the coefficient of IC on TEE are all positive and significant, which is the same result as the result from OLS model. This proves again that IC causes a significant positive influence on TEE.

In Column (5), the coefficient of spatial lag term, *ρ*, for the SDM model is −0.4588, which is negative and significant at the level of 5%, so this represents that the TEE has the significant negative spatial spillover effects in 284 cities. Namely, there is competition during the process of energy development in different regions, causing no harmonious development between various regions. The coefficient of IC and *W* × IC are 0.0918 and 0.7298, and both are positive and significant at the level of 5%. Thus, the IC can positively promote the TEE in the local and adjacent regions.

From the result of control variables, the level of economic development has positive effects on TEE in both local and adjacent regions; however, the effect on the local region is not significant based on the empirical. The result of the level of financial development in Column (5) inhibits the improvement of TEE in the local area and promotes that of TEE in the surrounding areas. The level of open-up is beneficial for the development of local TEE, and it is inhibitory for the adjacent areas, but both are not significant. The fiscal expenditure has positive effects on TEE in local areas and a negative impact on TEE in adjacent regions. The level of human capital also promotes the improvement of TEE in local and adjacent areas, but they are not significant from the empirical results. The level of transport infrastructure benefits the local regionally TEE improvement, and it has an inhibitory effect on the improvement of TEE from the adjacent areas, but this inhibitory effect is not significant from the empirical result.

Table 6 shows the results of direct effects, indirect effects, and total effects, and they are similar to the result from benchmark regression, which demonstrates the effectiveness of the result from benchmark regression.

**Table 6:** The result of direct effects, indirect effects, and total effects

	Direct effects	Indirect effects	Total effects
IC	0.0923**	0.4567**	0.5490**
	(2.101)	(2.015)	(2.441)
ED	0.0227	0.0739	0.0966**
	(0.954)	(1.493)	(2.301)
FD	−0.1250***	0.2114***	0.0864
	(−5.015)	(3.422)	(1.558)
*F*	0.4745	−5.6054*	−5.1309
	(1.19)	(−1.66)	(−1.48)
FE	0.0334***	−0.2242***	−0.1908**
	(2.673)	(−2.672)	(−2.247)
HC	0.0045	−0.0009	0.0037
	(0.294)	(−0.007)	(0.028)
TI	0.0229***	−0.0201	0.0028
	(3.095)	(−1.017)	(0.150)

*, **, and *** represent the significance at 10%, 5%, and 1%, respectively; *z*-value in the parentheses

The direct effects in Table 6 mean the overall effects of IC on TEE. The effects here contain the direct effect on TEE caused by TEE changes, which is the SDM estimation coefficient, and the “feedback effects” (Lou et al. 2021). The “feedback effect” is the further effect on TEE in the local area that is caused by the changes of the TEE in the adjacent areas, which are influenced by the IC in the local area (Ge et al. 2021). From Column (1) of Table 6, the results show that IC promotes the improvement of TEE, which every increase in 1% of IC would make a 0.0923 increase in TEE with the 0.0005 of “feedback effects.” Namely, IC can promote the local TEE by affecting the adjacent regions’ TEE.

The indirect effects in this paper mean the effects of TEE in adjacent regions caused by the IC from the local region. In other words, the indirect effects here represent the spillover effects of IC on TEE. Column (2) in Table 6 is the estimation result of the indirect effects. The results show that there was a 1% increase in IC in the local area, and the TEE in the surrounding cities increased by 0.4567. This further demonstrated the spillover effects of IC on TEE.

The coefficient of total effects, which is the sum of direct and indirect effects, in Column (3) Table 6 is 0.5490 and is significant at the significance level of 5%. Moreover, the estimation result of indirect and indirect effects for variables is close to the result in the benchmark regression.

### Results of the robustness test

The geographic distance weight matrix (W3) is replaced by the economic weight matrix (W1) to verify the robustness of the benchmark regression results. The result of the robustness test is shown in Table 7. The coefficient of IC is positive and significant to demonstrate that the IC can affect TEE in the local region again. From the result of SDM, in Column (3) in Table 7, the coefficient of *W* × IC is 0.2550 and significant at the level of 5%. These results are the same as the results from the benchmark regression. Furthermore, the significance level and direction of the control variable are roughly similar to the results from the benchmark regression. Thus, it is reasonable to believe that the benchmark regression result is robust.

**Table 7:** The result of the robustness test

TEE	(1)	(2)	(3)
	SAR	SEM	SDM
	W1	W1	W1
IC	0.0882**	0.0889**	0.0734*
	(2.060)	(2.076)	(1.700)
ED	0.0025	0.0022	0.0021
	(0.201)	(0.176)	(0.082)
FD	−0.0883***	−0.0876***	−0.0896***
	(−4.140)	(−4.130)	(−3.488)
*F*	0.7711**	0.7693**	0.7384*
	(2.004)	(2.00 )	(1.90)
FE	0.0364***	0.0363***	0.0298**
	(3.021)	(3.024)	(2.111)
HC	−0.0035	−0.0038	−0.0043
	(−0.228)	(−0.245)	(−0.277)
TI	0.0244***	0.0243***	0.0256***
	(3.562)	(3.555)	(3.494)
*W* × IC			0.2550**
			(2.313)
*W* × ED			−0.0084
			(−0.231)
*W* × FD			0.0208
			(0.482)
*W* × *F*			−0.2179
			(−0.22)
*W* × FE			0.0578*
			(1.714)
*W* × HC			−0.0652
			(−1.391)
*W* × TI			−0.0062
			(−0.365)
Observations	4544	4544	4544
Fixed effect	Yes	Yes	Yes
Time effect	Yes	Yes	Yes
Log-likelihood	−983.5044	−983.4658	−978.1745

*, **, and *** represent the significance at 10%, 5%, and 1%, respectively; *z*-value in the parentheses

### Results of the heterogeneity test

This paper differentiates the 284 cities in China into RBCs and NRBCs to have a further analysis of the regional differences between IC and TEE. Table 8 shows the results of this heterogeneity test.

**Table 8:** Results of heterogeneity test for resource-based cities and non-resource-based cities

	(1)	(2)
	RBC	NRBC
IC	0.1056**	0.0523
	(2.063)	(1.555)
*W* × IC	−0.1368**	0.1335**
	(−2.000)	(2.553)
Control variables	Yes	Yes
Fixed effect	Yes	Yes
Time effect	Yes	Yes
*ρ* or *λ*	0.1593***	0.0697***
	(6.898)	(3.104)
Log-likelihood	222.4706	804.3169
Observation	1824	2768

*, **, and *** represent the significance at 10%, 5%, and 1%, respectively; *z*-value in the parentheses

From Column (1) on Table 8, the coefficient of IC for RBC is 0.1056, which is positive and significant at the level of significance 5%, and the coefficient of *W* × IC is −0.1368, which is negative and significant at the level of 5%. From Column (2) on Table 8, the coefficient of IC for NRBC is 0.0612, which is positive but not significant, and *W* × IC is 1.3599, which is significant at the level of 5% and positive. Moreover, the coefficient of both the spatial lag terms (*ρ*) is positive and significant at the level of 1%.

This paper, set against the backdrop of the Chinese government’s commitment to transitioning from extensive economic growth towards achieving carbon neutrality, seeks to examine the relationship between IC and green energy efficiency at the urban level. It does so by employing spatial econometric methods and analyzing data from 284 Chinese cities, encompassing both RBCs and NRBCs. By utilizing spatial econometric tools and the super-efficiency SBM model with undesirable outputs, this paper addresses the series of questions initially posed: (1) How can the energy efficiency of China’s 284 prefecture-level cities be reasonably measured? (2) Can IC increase TEE? (3) Considering the time lag, does IC have a geographical spillover impact and regional variation on TEE? (4) Investigating the influence of time-related variations on the relationship between industrial agglomeration and green energy efficiency from the perspectives of RBCs and NRBCs cities.

To solve these questions, this paper collects the panel data from 284 prefecture-level cities in China, covering the period from 2005 to 2020. The data is sourced from the 2005–2020 China Urban Statistical Yearbook and the statistical yearbooks of the relevant cities and provinces.

The analysis conducted in this paper is based on the collected data, where the TEE efficiency is estimated using the super-efficiency SBM model with undesirable outputs. The measurement of IC is achieved through the entropy method. Additionally, the measurement methods for control variables are detailed in the relevant section of this paper. Furthermore, a spatial weight matrix is constructed to account for spatial relationships and interactions in the analysis.

The study evaluates the spatial correlations between TEE and IC using the Global Moran Index. The Moran Scatter Diagram is employed to visualize the results of the Local Moran Index. The positive spatial correlations observed for these variables provide the foundation for further analysis using spatial econometric methods. The study conducted a Hausman test, which indicates that using a fixed-effect model is more appropriate than the random model for the subsequent analysis. Additionally, the results of the LR and Wald tests demonstrate that the SDM model cannot be transformed into the SAR or SEM model.

According to the results of the tests, this study employed the SDM model to examine the spatial relationships between TEE, IC, and other control variables. Based on the empirical results, there is a clear positive spatial correlation between TEE and IC, indicating that IC has a positive impact on local TEE. Furthermore, significant spillover effects were observed between IC and the TEE of adjacent regions, suggesting that local IC can enhance TEE in neighboring areas. In addition, this paper conducted a robustness test by using an economic weight matrix instead of a geographic distance weight matrix. The results of the robustness test confirm the reliability of the benchmark regression results.

Lastly, this paper divides the 284 cities in China into RBCs and NRBCs to conduct a more detailed analysis of the regional variations in the relationship between IC and TEE. The findings reveal that the impact of IC on TEE, as well as its spillover effects, differs significantly between RBCs and NRBCs.

These findings offer valuable insights that can assist the Chinese government in various ways. They provide an understanding of the TEE at the geographical level in China and the spatiotemporal evolution of TEE differences from 2005 to 2020. Furthermore, they shed light on the impact of IC on green energy efficiency and its spatial spillover effects. The study also highlights the heterogeneous effects arising from RBCs and NRBCs. As a result, these results furnish essential theoretical support for the Chinese government’s efforts to promote TEE and IC. They aid in the formulation of tailored sustainable development plans for different regions and types of cities, facilitating the realization of carbon neutrality goals.

## Discussion

### Temporal and regional characteristics of TEE and IC

From Fig. 4, a discernible periodic pattern emerges, revealing that the trajectory of national TEE follows a “falling-first-then-rising” trend over five-year intervals. This temporal characteristic is consistent with the current research (Huang and Wang 2017). One plausible explanation for this phenomenon lies in China’s initial commitment to reducing energy intensity as outlined in the “11th Five-Year Plan.” Subsequently, there were ongoing efforts to reduce energy consumption and intensity not only during the “11th Five-Year Plan” but also continuously in the subsequent “12th Five-Year Plan” and “13th Five-Year Plan.” However, energy efficiency increased during the later stages of these plans due to the time conflicts between policy-making and implementation, leading to the observed inverted “U” shape.

The regional variation in TEE is observed as follows: TEE in the East > TEE in the West > TEE in the Central, with values of 0.6313, 0.6115, and 0.5074, respectively. This finding aligns with previous research (Yu 2020). The rationale behind this pattern can be attributed to the highly industrialized and economically developed coastal cities in eastern China. In contrast, the central region retains an extensive economic development model, often receiving less advanced production capacity from the East, resulting in more significant pollution in the Central. A similar situation occurs in the Western region, but what distinguishes it is that certain areas in the West still adhere to the original economic development model, contributing to the relatively higher TEE compared to the Central region.

The trajectory of IC from 2005 to 2020 reveals a consistent trend of growth and concentration in the central regions of China. The average IC was 2.7396, and it experienced an increase to 2.7658 in 2020. This can be attributed to several factors. Notably, China’s industrial structure has undergone significant transformations owing to the government-led upgrade of the industrial sector, with the proportion of the third industry continually increasing over the past decades, contributing to the rise in IC. Moreover, the relocation of industries, orchestrated by the government, played a role in this concentration trend. Industries from eastern cities were systematically transferred to the western and central regions, as supported by previous research (Wu et al. 2021). Additionally, the improvement in IC in the central region can be attributed to the spillover effects from the high IC areas in the East, as affirmed by Zhang et al. (2023b). Consequently, these factors converged to drive the concentration of IC in the central cities of China from 2005 to 2020.

### The impacts of IC on TEE and its spillover effects

According to the previous empirical analysis in the “Results” section, IC causes the positive effects of TEE in the local and adjacent areas. Thus, we will further discuss the reasons for this phenomenon in the following paragraphs.

The coefficient of 0.0918 indicates that IC has a positive impact on local TEE. This effect can be attributed to several factors. Firstly, IC benefits from economies of scale, leading to increased local TEE. Economies of scale result in cost savings and enhanced resource utilization, increasing the overall usage efficiency of factors and ultimately promoting TEE within the local region. Secondly, IC fosters technological development, which in turn positively affects local TEE. The heightened levels of IC create a more competitive market environment among firms. This competition compels companies to enhance their innovation and technology efficiency, reducing technological gaps and thus boosting local TEE. This point is supported by the findings of Cheng and Jin (2022). Thirdly, IC contributes to increased tax revenue for the local government. This additional income enables local governments to augment their fiscal expenditures (Li 2001), attracting more companies to the area and further enhancing local IC. Meanwhile, the government also may tend to improve the environment by adopting more strict local environmental regulations. These investments in the environment and improvements in local environmental regulations have a positive impact on TEE. The coefficient of 0.0332 for the positive effects of FE on local TEE reinforces this argument.

There are also spillover effects of local IC on the TEE in adjacent areas with a coefficient of 0.2550, which is larger than its impact in the local TEE. These spillover effects play a crucial role in disseminating sustainable and efficient knowledge and technology from the local area to the surrounding regions, subsequently enhancing the TEE of adjacent areas. Furthermore, the competitive dynamics induced by companies in adjacent areas, responding to the competitive pressure exerted by local firms, stimulate the development of their technology to increase efficiency. Consequently, both knowledge and technology spillover effects and competitive pressures contribute to the observed larger effects in adjacent areas compared to the local area. The indirect effects of IC, signifying a substantial positive result of 0.4567, further bolster this argument. Hence, these findings support Marshall’s positive externality theory of IC (Marshall 1920), indicating that IC generates positive externalities that benefit not only the local area but also its neighboring regions, thereby promoting overall industrial efficiency and sustainability.

The coefficient of *W* × FE and FE’s indirect effects are both significantly negative, with values of −0.3113 and −0.2242, respectively. This suggests that an increase in fiscal expenditure in the local region attracts companies and talents from the adjacent areas, resulting in a decrease in the knowledge and technology exchange between the local and adjacent regions. As a result, this has a negative impact on the TEE of the adjacent areas. The negative effects in the adjacent areas exceed the positive effects in the local areas, leading to an overall negative total effect of −0.1908.

Nonetheless, the FD is found to have negative effects on local TEE with a coefficient of −0.1270, corroborating the findings of Fan et al. (2023). The financial investment may inadvertently hasten the growth of IC within a local area, leading to an excessive concentration of resource factors in that area. This concentration, in turn, causes a crowding effect, ultimately harming the local TEE. Conversely, the coefficients of *W* × FD and FD’s indirect effects are both notably positive, standing at 0.2532 and 0.2114, respectively. This may be attributed to the positive influence of increased FD in the local area on the adjacent areas’ IC. This could lead to more rational resource allocation and foster an aggregation effect that positively impacts TEE.

IC has varying effects and spatial spillover between RBCs and NRBCs. The heterogeneity test reveals that in RBCs, IC has a positive impact on local TEE with a coefficient of 0.1056. This can be attributed to the economies of scale and technological advancements resulting from IC. However, this same local IC has a negative effect on adjacent areas’ TEE, with a coefficient of −0.1368. Technological improvements in the local area may not trickle down to the adjacent area if the technology is specific to certain natural resources, as noted by Chen et al. (2019), leading to inefficient technology spillover and negative impacts on the TEE in adjacent areas. For example, Datong and Zhangjiakou are adjacent RBCs. Zhangjiakou has abundant metal minerals, making the technological improvement in metal minerals useless for Datong.

Nevertheless, IC has positive spillover effects in the adjacent NRBCs with a coefficient of 0.1335. Unlike RBCs, NRBCs can benefit from IC without being constrained by specific natural resources. This allows for the free flow of technology and knowledge in the adjacent areas. As a result, the effects of IC may extend beyond the local area and have a wider impact. This helps to explain why the effects of IC on local TEE may not be significant, but they do have positive effects on TEE in adjacent areas. This discussion further supports the notion of knowledge spillover effects (Döring and Schnellenbach 2006).

## Conclusion and policy recommendations

IC is an inevitable spatial phenomenon during China’s economic development and significantly impacts cities’ energy efficiency. This paper utilizes panel data from 284 Chinese cities to measure TEE using the super-efficiency SBM model with the undesirable outputs model. Based on this, the SDM is employed to test the influence of IC on TEE and its spillover effects. The main conclusions of this study are as follows:

Firstly, the average TEE of the 284 Chinese cities from 2005 to 2020 is 0.5834, which indicates room for improvement. When considering the temporal scale, TEE exhibits a fluctuating trend of initially declining and then rising. In terms of regional distribution, the East has the highest mean TEE (0.6313), followed by the West (0.6115), and the Central regions (0.5074). The analysis also reveals a growing and concentrating trend of IC in the central regions, increasing from 2.7396 to 2.7658 between 2005 and 2020.

Secondly, the study demonstrates that IC has a positive impact on local TEE, as evidenced by a coefficient of 0.0918. Additionally, there is a positive correlation between fiscal expenditure and local TEE, indicated by a coefficient of 0.0332. These findings further support the conclusion that increased tax income resulting from IC plays a crucial role in driving improvements in local TEE. Conversely, financial development negatively affects local TEE, with a coefficient of −0.1270.

Thirdly, there are spillover effects of local IC on TEE in adjacent areas, indicated by a coefficient of 0.2550, and the indirect effect of IC is measured at 0.4567, which is larger than its impact on local TEE. Moreover, FE also shows spillover effects on adjacent TEE, with notably negative coefficients for *W* × FE (−0.3113) and FE’s indirect effects (−0.2242). However, it is noteworthy that the coefficients for *W* × FD (0.2532) and FD’s indirect effects (0.2114) are significantly positive, indicating positive spillover effects on adjacent TEE.

Finally, it is essential to highlight that the impacts of IC on TEE and its spillover consequences vary between RBCs and NRBCs. In RBCs, IC has a positive influence on local TEE, with a coefficient of 0.1056. However, this positive impact is not observed in adjacent areas; instead, it exerts a negative effect, with a coefficient of −0.1368. Conversely, in NRBCs, IC enhances TEE in adjacent cities, as evidenced by a coefficient of 0.1335, while its impact on local cities’ TEE is found to be statistically insignificant.

Based on the above conclusion, we offer some policy recommendations and research implications:

To address the volatility of national TEE and narrow the gap between regional TEE, several measures should be considered. From a national perspective, the government should draw lessons from past experiences and streamline the policy-making and implementation processes to minimize time conflicts and reduce the volatility of national TEE over 5 years. From a regional standpoint, the eastern area should receive support to further increase its TEE. Additionally, areas in the East with high TEE should provide targeted assistance to areas in the West and Central regions with low TEE. This assistance could involve optimizing industrial structures and upgrading industries. For example, transferring certain industries to balance the industrial structures to the region with low TEE to increase IC and improves the TEE, thus narrowing the TEE gap between different areas.

The government should also prioritize increasing the local IC while harnessing its positive effects on local TEE and mitigating any negative impacts. Firstly, the government could increase fiscal expenditure to attract nonlocal companies by investing in local infrastructure, providing interest-free loans, and improving the local business environment. These efforts would stimulate IC and enhance TEE. Secondly, the government should enhance local environmental regulations, such as offering preferential policies to environmentally friendly firms or establishing special funds to accelerate the development of local green technology. This would guide local firms towards higher TEE practices. Thirdly, the government should carefully manage resource allocation to mitigate crowding effects as IC increases. It is crucial to allocate resources reasonably to prevent potential inefficiencies.

The government should harness the spillover effects of IC on adjacent TEE through knowledge exchange, local investment, and fiscal strategies to facilitate balanced development. Firstly, the government should actively promote knowledge and technology exchange between local areas and neighboring regions. This can be achieved by organizing technical seminars and facilitating knowledge-sharing meetings to enhance the dissemination of expertise and technology between different locations, ultimately augmenting the spillover effects of knowledge and technology. Secondly, the government can play a pivotal role in stimulating local investments and capitalizing on the positive spillover effects in adjacent areas. This support should be directed towards the responsible development of adjacent IC, taking into consideration the trade-off between its potential negative impacts on the local area and the positive spillover effects on neighboring regions. Lastly, when local governments intend to increase fiscal expenditure to attract more businesses and industries, they must be mindful of the potential negative spillover effects on adjacent TEE and adopt measures such as fiscal transfers to compensate for the negative repercussions in adjacent areas to simultaneously promote their TEE development. This approach ensures a more equitable and balanced growth pattern across regions.

The government should implement sustainable development plans to leverage the effects of IC on local TEE and its spillover effects in RBCs and NRBCs. In RBCs, the government should decrease the dependency on specific natural resources to prevent knowledge and technology from being confined to specific areas. For instance, the government should develop industries that do not rely on specific local resources to diversify the local economy. This approach will increase local IC and TEE while mitigating negative spillover effects on adjacent areas’ TEE. In NRBCs, the government should attract specific industries to the local area and establish locally advantageous and characteristic industries. This will allow the positive effects of IC to remain localized while maintaining positive spillover effects on adjacent areas.

In this study, there are some limitations to this paper, and it is expected to be improved by further future research. Firstly, using the country-level city data might analyze the effects of IC on TEE in more detail to make more interesting observations. Secondly, the data can be updated once the new data is out, and it is valuable to observe the novel changes in TEE and IC. This paper hopes to promote more research on IC and TEE to develop some policies to improve both the development of IC and TEE.

## Data Availability

The data that support the findings of this study are available from www.cnki.net.
